# Augmenting medical image specific foundation model with classical radiomic signatures for improved nasopharyngeal carcinoma stage classification

**DOI:** 10.3389/fmedt.2026.1863203

**Published:** 2026-07-15

**Authors:** Yu Li, Jiacheng Liu, Xin Wen, Xiaomin Niu, Lai Wei, Junjie Yuan, Zhiqiang Wang, Rong Na

**Affiliations:** 1 Department of Otolaryngology-Head and Neck Surgery, The Eighth Affiliated Hospital, Sun Yat-Sen University, Shenzhen, China; 2 Department of Urology, Ruijin Hospital, Shanghai Jiao Tong University School of Medicine, Shanghai, China; 3 Department of Orthopedics, Shanghai Fengxian District Central Hospital, Shanghai Jiao Tong University Affiliated Sixth People's Hospital South Campus, Shanghai, China; 4 Department of Surgery, School of Clinical Medicine, LKS Faculty of Medicine, The University of Hong Kong, Hong Kong, Hong Kong SAR, China; 5 Department of Surgery, Queen Mary Hospital, Hong Kong, Hong Kong SAR, China

**Keywords:** cancer staging, deep learning, nasopharyngeal carcinoma, radiomics, vision transformer

## Abstract

**Background:**

Accurate clinical staging is the critical process of prognosis and treatment planning for nasopharyngeal carcinoma (NPC). Conventional radiomics is limited to hand-crafted features that may overlook complex patterns, while generic deep learning models often lack domain specificity. Currently, SAM-Med3D, a vision transformer (ViT)-based medical model excels at capturing global volumetric context. We hypothesized that fusing radiomic and SAM-Med3D feature-extraction would yield synergistic value. This study aims to construct an MRI-based fusion model for NPC stage classification (Stage I, II and III vs. Stage IVa) and benchmark its performance against experienced experts.

**Methods:**

We retrospectively analyzed 264 patients with non-metastatic nasopharyngeal carcinoma, partitioned into training (*n* = 198) and independent validation (*n* = 66) cohorts. MRI sequences (T1WI, T2WI, CE-T1WI) were utilized to extract high-dimensional features by two distinct paradigms: hand-crafted radiomics and deep features from SAM-Med3D. An early-fusion dataset (SAM-PR) integrating both feature types was also constructed. Following LASSO-based dimensionality reduction, five machine learning classifiers (LR, SVM, RF, XGB, LGBM) were developed for staging. Model performance was evaluated using the area under the curve (AUC) and benchmarked against the independent, blinded staging of two senior otolaryngologists. Optimism-Corrected Bootstrap analysis was applied to evaluate the reliability of AUC, and the learning curve was conducted to avoid overfitting.

**Results:**

LASSO regression identified an optimal fusion set of 17 features (9 hand-crafted and 8 deep-learned). Among all 15 constructed DL models, the fusion-based LR yielded the optimal performance (AUC = 0.760, optimism-corrected AUC of 0.836), and the fusion approaches consistently yielded higher AUCs than individual feature approaches. Compared to human experts, our optimal model achieved a more balanced performance (Sensitivity: 73.91% vs. 88%; Specificity: 69.77% vs. 26%–30%) and a higher F1-score (0.615 vs. 0.353–0.400).

**Conclusions:**

The integrated model, combining conventional radiomics with SAM-Med3D features, consistently showed higher accuracy than its unimodal counterparts across five distinct algorithms (LR, SVM, RF, XGB, LGBM) for NPC stage I, II, III and stage IVa differentiation. All five fusion models demonstrated comparable discriminative ability compared to experienced experts. These findings suggest that hybrid feature frameworks can serve as powerful objective tools in NPC staging.

## 1 Introduction

Nasopharyngeal carcinoma (NPC) is characterized by a distinct geographical distribution and is particularly prevalent in East and Southeast Asia (1). In Southern China, it ranks the highest in incidence among head and neck tumor (2). Accurate clinical staging is the most critical factor for determining prognosis and guiding therapeutic strategy. For instance, among locoregionally advanced NPCs, patients with stage IVa disease have significantly inferior overall survival compared to those with stage III disease, with a corresponding hazard ratio of 1.702 (3).

Magnetic Resonance Imaging (MRI) is currently the optimal approach for staging NPC due to its superior soft-tissue contrast (4, 5). Computer-vision, by extracting and analyzing digital features to understand the image, has added substantial value to the MRI image interpretation (6, 7). One conventional method is PyRadiomics (8), an open-source framework for extracting radiomic features from medical images. It quantifies tumor phenotype by calculating a large set of pre-defined mathematical descriptors. While radiomic signatures have shown promise, they are limited to pre-defined patterns and may fail to capture the full spectrum of hierarchical and contextual information within an image.

Concurrently, computer-vision and deep-learning (DL) have been revolutionized by foundation models, which are large-scale models pre-trained on vast datasets. Vision Transformers (ViT) (9) architecture is the dominant force with outstanding performance, processing images by using a self-attention algorithm (10) to learn global relationships between image patches. The Segment Anything Model (SAM) is a prominent FM that leverages a ViT encoder to achieve remarkable generalization. SAM-Med3D, a novel adaptation of this architecture, has exhibited an impressive performance on medical imaging tasks by re-engineering the encoder to directly process 3D volumetric data (11).

To date, PyRadiomics features and ViT embeddings have been explored largely in isolation. We hypothesize that these two feature sets are not redundant but are highly complementary. In this study, we aim to (1) investigate the synergistic potential of fusing these two distinct feature-extraction paradigms, (2) construct an MRI-based DL model for the task of differentiating stage I, II, III and stage IVa NPC, and (3) benchmark our optimal DL model with human experts in this specific task.

## 2 Methods

### 2.1 Dataset description

Data was acquired from a cohort of 277 patients with histopathologically confirmed NPC who underwent multi-parametric MRI, including T1-weighted (T1WI), T2-weighted (T2WI), and contrast-enhanced T1-weighted (CE-T1WI) sequences from a latest Chinese population-based opensource dataset (5). Imaging data was acquired from six different MR scanners (GE Discovery MR750w 3.0T and Philips Achieva 1.5T systems). To ensure consistency and to minimize variability across the different scanners, a rigorous standardization and calibration protocol was implemented. All MRI scanners were calibrated before the study and subjected to regular quality control. Procedures included geometric verification via standardized phantoms, signal intensity normalization to ensure temporal consistency, and magnetic field optimization to minimize artifacts. These measures guaranteed the stability of spatial resolution and signal brightness across the entire imaging period. All the segmentations of region-of-interest (ROI) were performed manually by two experienced diagnostic radiologists (each with over 10 years of experience) independently. Inter-observer reliability was assessed using Dice similarity coefficients and Jaccard indices on a subset of 30 patients. The results showed median Dice scores > 0.90 and Jaccard scores > 0.80 across all sequences (T1WI, T2WI, CE-T1WI), providing quantitative evidence of the high segmentation accuracy between the two radiologists (5). It is important to note that all patients in this dataset were staged according to the 8th Edition AJCC/UICC staging system (12). In this edition, unlike the 9th Edition, Stage IVa is defined as T4N0-2M0 or T_any_N3M0, strictly excluding distant metastasis.

### 2.2 Patient cohort and data division

**Figure 1:**
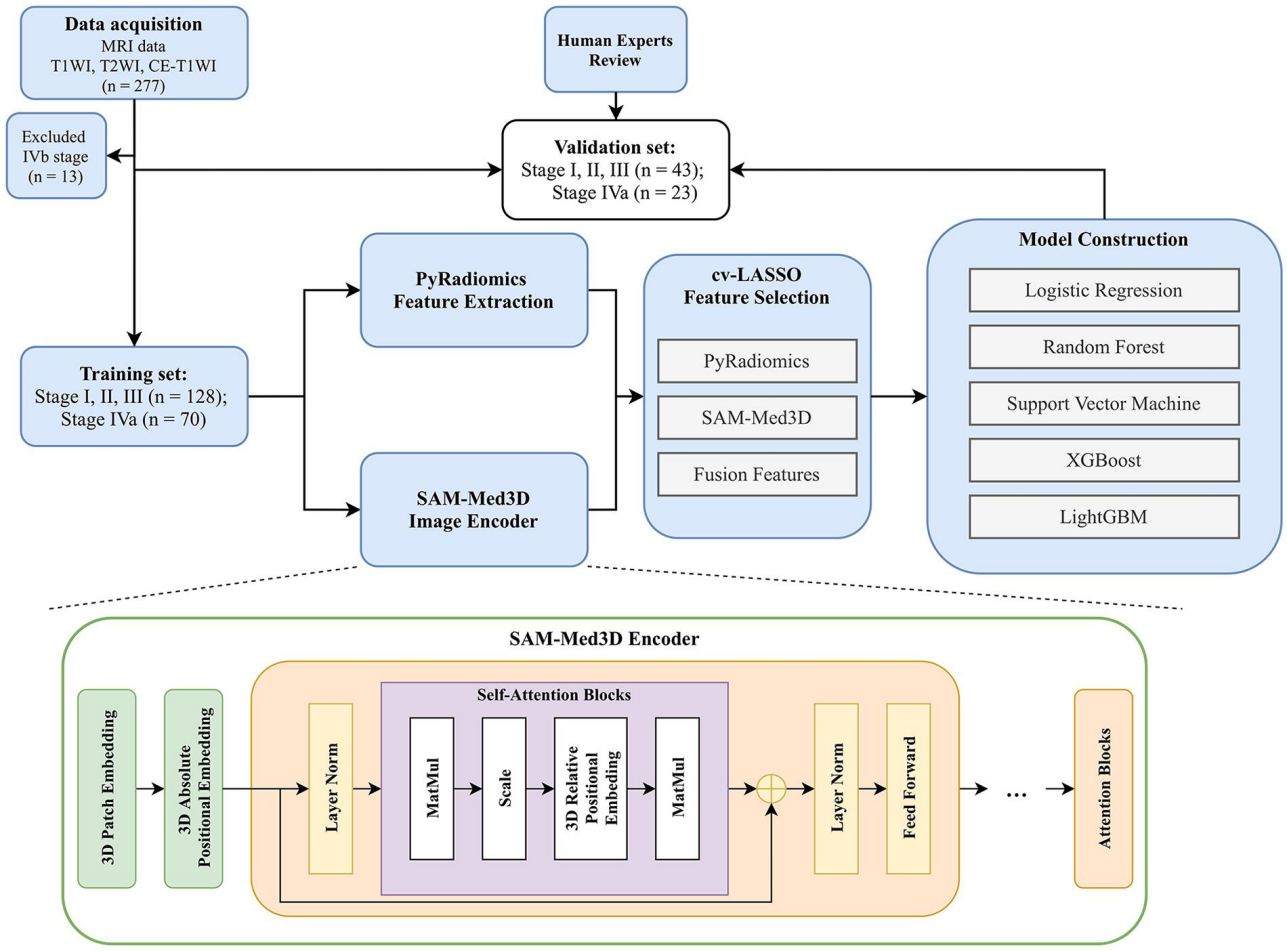
The workflow of this study. The initial cohort (*n* = 277) was screened to exclude Stage IVb cases (*n* = 13). The remaining patients were split into a Training set (*n* = 198) and a Validation set (*n* = 66), with stages categorized into early-to-locally advanced (I, II, III) and advanced (IVa). Features were extracted using two parallel pipelines: traditional handcrafted radiomics via PyRadiomics and deep learning-based embeddings via the SAM-Med3D Image Encoder. The lower panel details the encoder structure, utilizing 3D Patch and absolute positional embeddings followed by Transformer-based self-attention blocks. These blocks incorporate layer normalization, 3D relative positional embedding, and feed-forward networks to capture spatial-contextual information. Feature dimensionality was reduced using cv-LASSO across three categories: PyRadiomics, SAM-Med3D, and Fusion features. Final predictive performance was evaluated across five machine learning classifiers (LR, RF, SVM, XGBoost, and LightGBM).

The workflow was shown in Figure 1.

Patients diagnosed with stage IVb disease (*n* = 13) were excluded from the analysis. The remaining 264 patients were randomly partitioned into a training set (*n* = 198) and a validation set (*n* = 66). The training set comprised 128 patients with stage I–III disease and 70 patients with stage IVa disease, while the validation set included 43 and 23 patients from these stage groups, respectively. Patient demographics were described in Table 1.

**Table 1:** Demographics of the dataset included in this study.

Parameters	All participants (*n* = 264)	Training group (*n* = 198)	Test group (*n* = 66)	*p* value (Training group vs. Test group)
Age (years)	50.3 ± 12.9	49.8 ± 12.7	51.7 ± 13.4	0.315^a^
Sex				0.334^b^
Male	194 (73.48%)	142 (71.72%)	52 (78.79%)	
Female	70 (26.52%)	56 (28.28%)	14 (21.21%)	
T				0.270^b^
T1	15 (5.68%)	10 (5.05%)	5 (7.58%)	
T2	62 (23.48%)	52 (26.26%)	10 (15.15%)	
T3	112 (42.42%)	80 (40.40%)	32 (48.48%)	
T4	75 (28.41%)	56 (28.28%)	19 (28.79%)	
N				0.545^b^
N0	25 (9.47%)	19 (9.60%)	6 (9.09%)	
N1	82 (31.06%)	66 (33.33%)	16 (24.24%)	
N2	127 (48.11%)	91 (45.96%)	36 (54.55%)	
N3	30 (11.36%)	22 (11.11%)	8 (12.12%)	
Stage^c^				0.984^b^
I	5 (1.89%)	4 (2.02%)	1 (1.52%)	
II	26 (9.85%)	20 (10.10%)	6 (9.09%)	
III	140 (53.03%)	104 (52.53%)	36 (54.55%)	
IVa	93 (35.23%)	70 (35.35%)	23 (34.85%)	

a *T*-test.

b Chi-square test.

c Staged according to the 8th edition UICC/AJCC staging system.

### 2.3 Feature engineering

**Figure 2:**
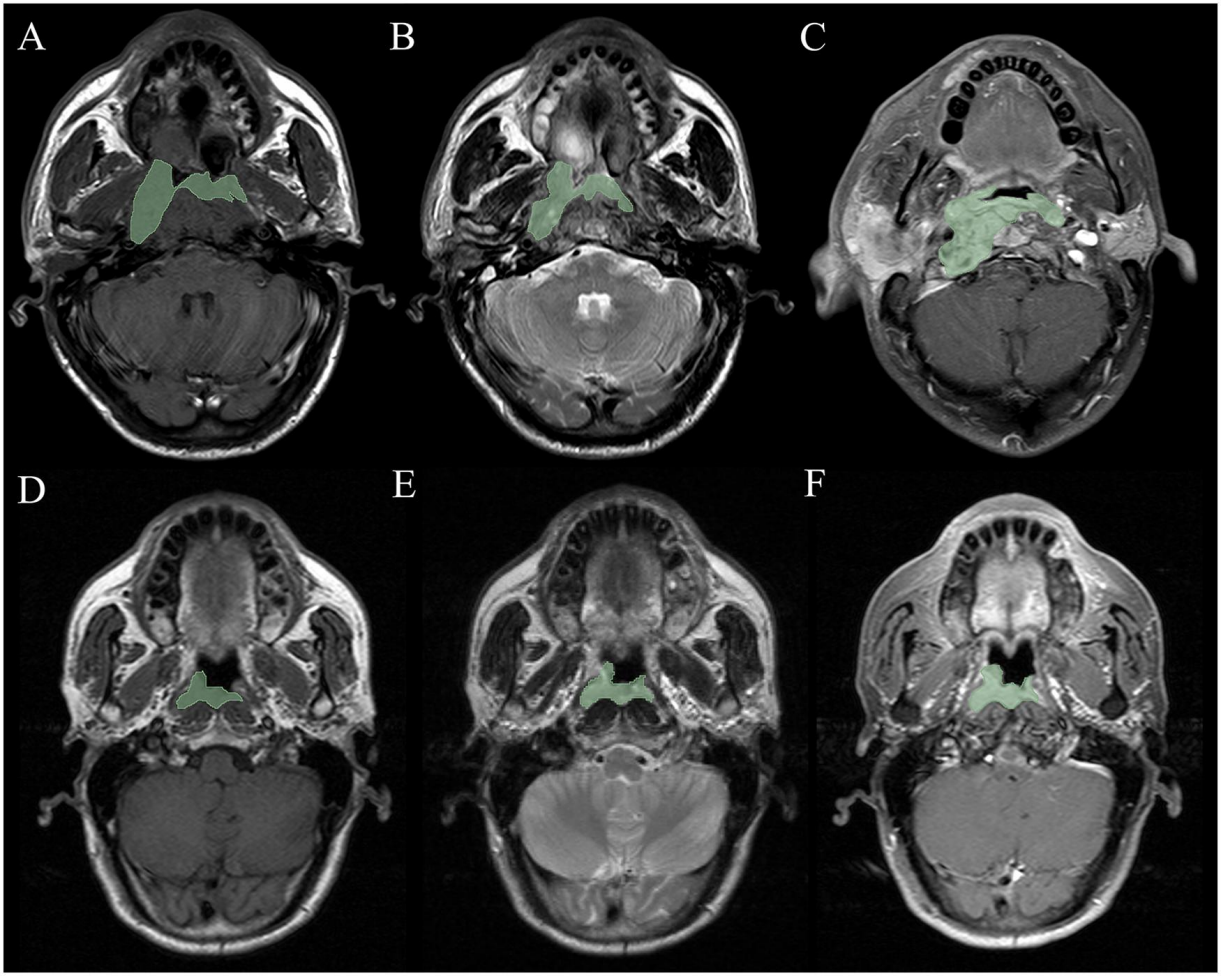
MRI showcase. The figure showcases two patients’ MRI images including T1WI **(A,D)**, T2WI **(B,E)**, and CE-T1WI **(C,F)** sequences acquired from the opensource dataset (5), which were processed by the manual ROI delineation respectively.

We developed and compared three distinct feature sets derived from the primary ROI on each patient's MRI T1WI, T2WI and CE-T1WI series (Figure 2).

#### 2.3.1 Radiomic feature extraction via PyRadiomics

Radiomic feature extraction was performed using the “PyRadiomics” (v3.7) package (8) in Python, with “SimpleITK” (13) as the image processing backend, adhering to the Image Biomarker Standardization Initiative (IBSI) (14).

Prior to extraction, a standardized preprocessing protocol was applied. All images were resampled to an isotropic voxel spacing of 1 × 1 × 1 mm^3^ using B-spline interpolation. Subsequently, image intensities were normalized, and a fixed bin width of 25 was used for intensity discretization. A mask correction algorithm was also enabled to ensure geometric consistency between the image and mask volumes.

Features were extracted from both the original preprocessed images and from the derived, filtered images. Two types of filters were applied: a Laplacian of Gaussian (LoG) filter (with sigma values of 1.0, 3.0, and 5.0 mm) and a full 3D Wavelet decomposition. In total, seven classes of radiomic features were calculated: 3D Shape, First-Order Statistics, Gray Level Co-occurrence Matrix (GLCM), Gray Level Run Length Matrix (GLRLM), Gray Level Size Zone Matrix (GLSZM), Gray Level Dependence Matrix (GLDM) and Neighbouring Gray Tone Difference Matrix (NGTDM).

#### 2.3.2 Deep feature extraction via SAM-Med3D

We also extracted deep features using the pre-trained SAM-Med3D model (11). Each 3D image was fed into the model's image encoder to generate a high-dimensional 3D feature map. This map represents a rich, learned embedding of spatial and contextual information.

Before extraction, we preprocessed the medical image. First, the ground-truth segmentation mask was downsampled using nearest-neighbor interpolation to preserve the discrete boundaries of the ROI. Next, the image volume's voxel intensities were normalized to a [0, 1] range using min-max scaling. Both the normalized image and its aligned mask were then resized to the SAM-Med3D model's required input dimension of 128 × 128 × 128. This resizing was performed using trilinear interpolation for the continuous image data and nearest-neighbor interpolation for the categorical mask data. Finally, the processed volumes were converted to PyTorch tensors and then passed to the pre-trained SAM-Med3D for feature extraction. Leveraging the weights trained on a large dataset, we mapped each MRI series to a 384-dimensional “SAM feature” vector.

### 2.4 Model construction

#### 2.4.1 Feature selection for three sets

The early-fusion approach, connecting all features from different modalities into a single feature vector, was employed in this study. Three different feature sets, PyRadiomics, SAM-Med3D and SAM-PR (the fusion feature set with both PyRadiomics and SAM-Med3D extracted features) were applied for the subsequent model construction process. All extracted features were standardized using a *Z*-score transformation fitted strictly on the training cohort; the resulting scaling parameters were then independently applied to the validation cohort to prevent information leakage. Subsequently, to reduce high-dimensionality, we applied L1 (cv-LASSO) regularization with five-fold stratified cross-validation exclusively to the standardized training data.

Finally, three distinctive sets with selected features were used for further model construction for the classification task (Stage I, II and III vs. Stage IVa).

#### 2.4.2 Five different machine-learning algorithms

With three feature sets, we constructed and evaluated five different machine learning models, Logistic Regression (LR), Support Vector Machine (SVM), Random Forest (RF), XGBoost (XGB) and Light Gradient Boost Machine (LGBM).

To find the optimal hyperparameters for each model, we employed a grid search with five-fold stratified cross-validation on the training data. The primary metric for model optimization and selection was the Area Under the Receiver Operating Characteristic Curve (AUC). To address the class imbalance between Stage ≤ III and Stage IVa patients, all models incorporated class weight balancing. The performance of each optimized model was independently validated on the unseen test set. The primary evaluation metric was the AUC.

### 2.5 Human doctor evaluator

We invited two otolaryngologists to review and re-stage the patients in the test set independently. These two experts were senior otolaryngologists, each possessing over 15 years of specialized clinical experience in the diagnosis and management of nasopharyngeal carcinoma. To ensure an unbiased evaluation, both clinicians were strictly blinded to the patients' clinical records, histopathological outcomes, and the predictions generated by the AI models. They were provided with the identical MRI sequences (T1WI, T2WI, and CE-T1WI), age, and gender utilized by the computational framework and were instructed to perform binary staging (Stage ≤ III vs. Stage IVa) according to the UICC/AJCC 8th edition guidelines (12).

### 2.6 Statistical analysis

Chi-square test was used to compare categorical variables and an independent *T*-test was used for continuous variables. Receiver operating characteristic (ROC) curves and AUC, F1 score, accuracy, sensitivity, and specificity were used to assess the performance of the predictive model. To rigorously evaluate potential overfitting and assess data saturation relative to the selected feature space, learning curve analysis was performed by tracking the convergence of training and cross-validation scores across incremental sample sizes. Additionally, to quantify potential performance inflation and ensure robust internal validity, an optimism-corrected bootstrap analysis with 1,000 resamples was conducted to calculate the model's internal optimism and derive the overfitting-adjusted AUC. The Delong test was used to compare AUCs and to calculate 95% confidence interval (CI) correspondingly. McNemar's test was applied to compare the models' performances against human readers'. To understand the internal logic of the trained models, to improve the interpretability and reliability of our model, and to identify the most critical predictors, we performed a *post-hoc* interpretability analysis using SHAP (SHapley Additive exPlanations) (15). In this study, *p*-value < 0.05 was considered statistically significant. All statistical analyses were performed using Python 3.10.19. The authors have completed the TRIPOD reporting checklist (16).

## 3 Results

### 3.1 Demographics of dataset

The final study cohort consisted of 264 patients with histopathologically confirmed nasopharyngeal carcinoma. The mean age of the entire cohort was 50.3 ± 12.9 years, with a male predominance (194/264, 73.48%). Regarding disease severity, 93 (35.23%) patients were in the stage IVa group, and 171 (64.77%) patients were categorized in the ≤stage III group.

The cohort was randomly partitioned into a training set (*n* = 198, 75%) and an independent test set (*n* = 66, 25%). No significant differences were observed between the training and test sets regarding age (49.8 vs. 51.7, *p* = 0.315), sex (*p* = 0.334), T-stage (*p* = 0.270), N-stage (*p* = 0.545), or overall AJCC stage (*p* = 0.984). Detailed patient demographics and clinical characteristics are summarized in Table 1.

### 3.2 Feature selection and dimensionality reduction

**Figure 3:**
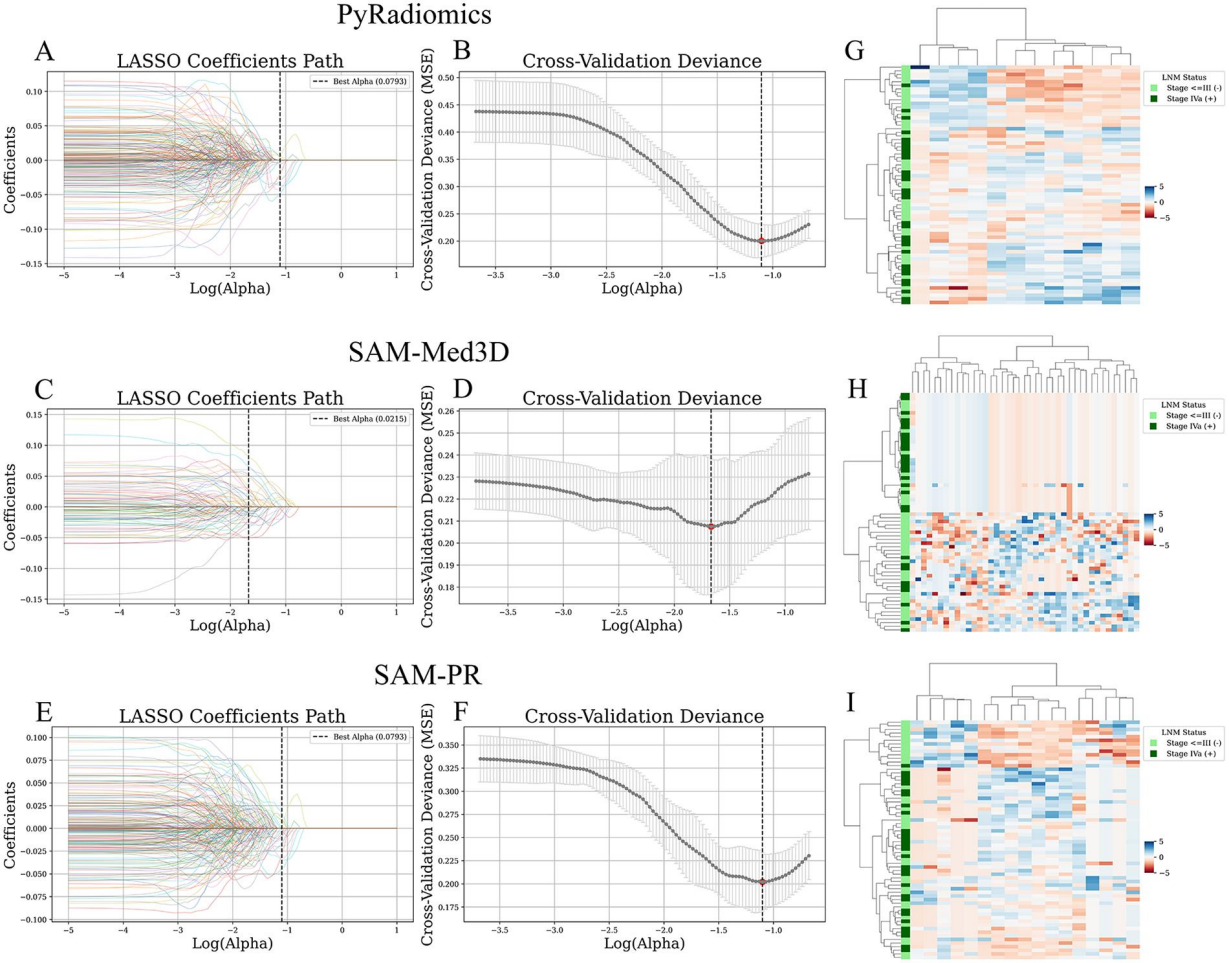
Feature selection analysis. This figure illustrates the dimensionality reduction and discriminatory power of the three distinct feature sets. **(A–F)** Least Absolute Shrinkage and Selection Operator (LASSO) regression analysis with 5-fold cross-validation. The left column **(A,C,E)** displays the coefficient profiles of the features as the regularization parameter [*λ*, plotted as Log(Alpha)] varies. The right column **(B,D,F)** plots the cross-validation deviance (Mean Squared Error) against Log(Alpha). The vertical dashed lines indicate the optimal *α* value that minimizes the error (marked by the red dot). **(G–I)** Unsupervised hierarchical clustering heatmaps of the selected feature signatures for **(G)** PyRadiomics, **(H)** SAM-Med3D, and **(I)** Fusion sets. Columns represent individual patients, and rows represent the selected features. The color scale indicates the standardized *Z*-score of feature expression. The patient class labels are annotated on the left (Light green: Stage ≤ III; Dark green: Stage IVa).

The optimal parameter sets were determined via 5-fold cv-LASSO using the minimum binomial deviance criterion (Figure 3 and Supplementary Table S1). The optimal regularization parameter (*α*) for each set was chosen by identifying the value that minimized the cross-validation MSE (mean squared error), as shown by the red dot in Figures 2B, D,And 2F.

The optimal *α* for PyRadiomics set was found to be −0.0793. The selection process yielded a final subset of 12 predictive features (3 from T1WI, 5 from T2WI and 4 from CE-T1WI). This set was predominantly composed of textural features, including GLCM (5 features), GLDM (3 features), NGTDM (1 feature) and GLSZM (1 feature), derived from Wavelet and LoG-filtered images (Supplementary Table S1). For the SAM-Med3D approach, a subset of 41 features was selected with the optimal *α* of 0.0215. And the fusion set yielded a compact signature of 17 features (9 from PyRadiomics, and 8 from SAM-Med3D) at an optimal *α* of 0.0793.

We visualized the discriminatory power of these final selected feature sets using clustering heatmaps (Figures 3G–I). PyRadiomics set demonstrated weak discriminatory power (Figure 3G). In contrast, the SAM-Med3D feature set and SAM-PR feature set showed much better separability. The unsupervised clustering algorithm substantially grouped the patients into two large, distinct clusters that aligned with the ground-truth class labels (Figures 3H,I).

### 3.3 Model performance

We trained and tested all the 15 models, five distinct machine learning algorithms (LR, SVM, RF, XGB, and LGBM) using the three engineered feature sets.

#### 3.3.1 Superiority of the fusion feature extraction approach

**Figure 4:**
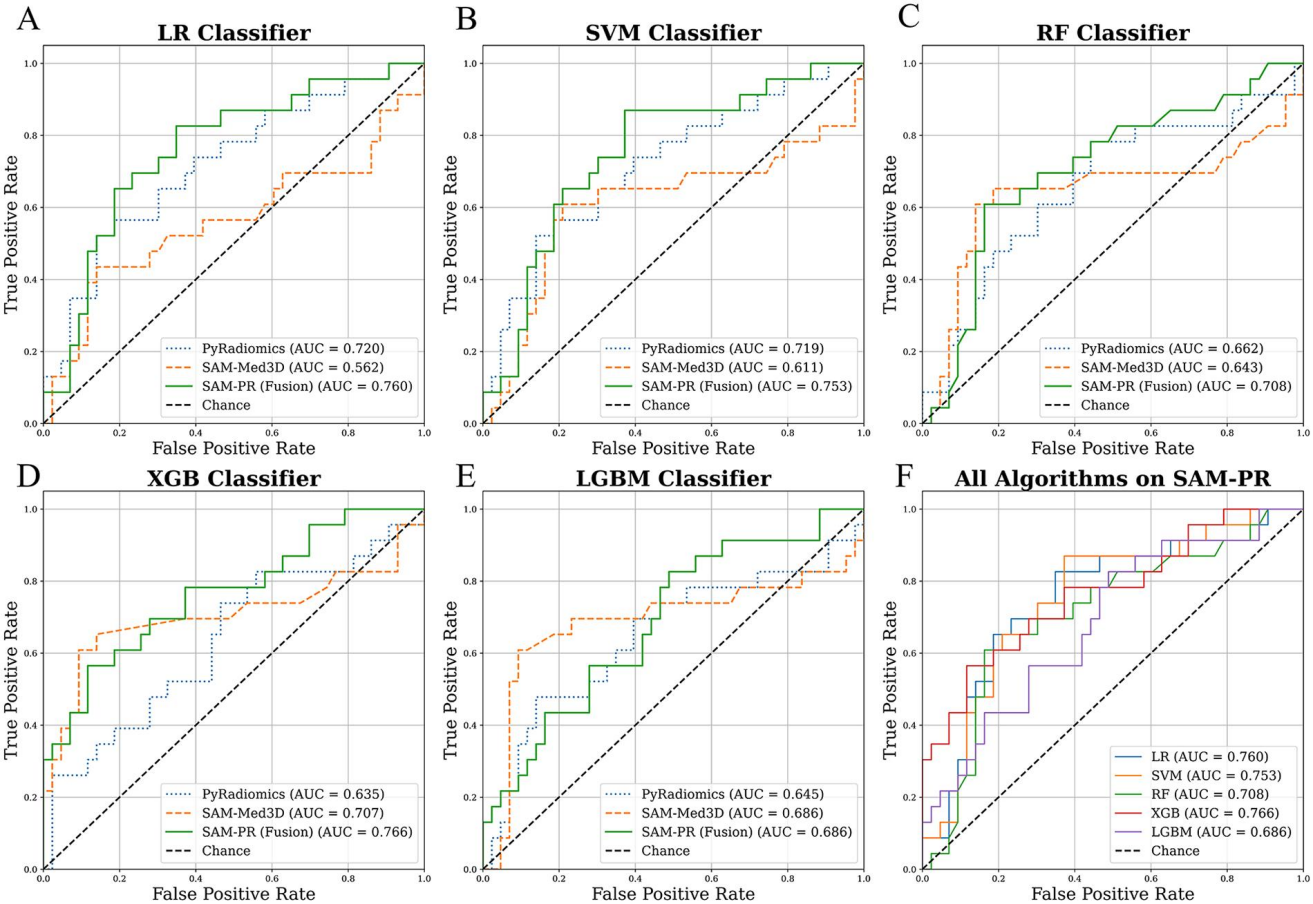
Receiver operating characteristic (ROC) analysis on the independent test Set. **(A–E)** Comparison of discriminative performance among the three feature sets—PyRadiomics, SAM-Med3D, and SAM-PR—across five distinct machine learning classifiers: **(A)** Logistic Regression, **(B)** Support Vector Machine, **(C)** Random Forest, **(D)** XGBoost, and **(E)** LightGBM. **(F)** Comparative performance of the five algorithms utilizing the optimal Fusion (SAM-PR) feature set.

The SAM-PR fusion feature set, which integrated hand-crafted radiomics with deep-learned features, consistently demonstrated superior performance compared to either PyRadiomics or SAM-Med3D approach. As illustrated in the ROC curves (Figures 4A–E), the SAM-PR models achieved the highest AUC in all the five algorithms tested.

#### 3.3.2 Evaluation of classifiers

In the initial hold-out validation among models utilizing the fusion feature set, the XGB classifier yielded the highest apparent discriminative ability (AUC = 0.766, 95% CI: 0.642–0.891), followed closely by the LR (AUC = 0.760, 95% CI: 0.637–0.884) and SVM (AUC = 0.753, 95% CI: 0.629–0.878) (Figure 4F and Table 2). However, to rigorously assess the risk of overfitting on our relatively constrained cohort, we conducted a *post-hoc* learning curve and optimism-corrected bootstrap analysis. Learning curves revealed that the XGB model exhibited severe overfitting tendencies, characterized by a generalization gap between training and cross-validation scores. In contrast, the learning curve for the regularized LR model on the fusion set demonstrated excellent convergence and early data saturation with a negligible generalization gap (Supplementary Figure S3). Furthermore, the 1,000-iteration bootstrap analysis confirmed the exceptional internal stability of the LR fusion model, yielding a highly robust optimism-corrected AUC of 0.836 (apparent AUC = 0.849; mean optimism = 0.013, Supplementary Table S3). Consequently, the LR model was prioritized as the optimal and most generalizable clinical pipeline. Additionally, the effectiveness of the feature sets was algorithm-dependent. Tree-based models, including XGB and LGBM, showed a preference for the deep-learning (SAM-Med3D) features to PyRadiomics features, yielding higher yet not significant AUCs (SAM-Med3D vs. PyRadiomics: XGB, 0.707 (95% CI: 0.548–0.866) vs. 0.635 (95% CI: 0.487–0.783), *p* = 0.5176; LGBM, 0.686 (95% CI: 0.522–0.850) vs. 0.654 (95% CI: 0.492–0.798), *p* = 0.7024). In contrast, the linear (LR) and kernel-based (SVM) models were more effective when trained on the engineered PyRadiomics features than on SAM-Med3D features, which resulted in a numerically better performance (PyRadiomics vs. SAM-Med3D: LR, 0.720 (95% CI: 0.588–0.852) vs. 0.562 (95% CI: 0.398–0.726), *p* = 0.0963; SVM, 0.719 (95% CI: 0.586–0.852) vs. 0.611 (95% CI: 0.446–0.775), *p* = 0.1445).

**Table 2:** Performance matrix of different models based on three feature sets.

Algorithm	Feature set	AUC (95% CI)	Accuracy (95% CI)	Sensitivity (95% CI)	Specificity (95% CI)	(95% CI)	*p* value (vs. PyRadiomics)	*p* value (vs. SAM-Med3D)
Test set								
LR	PyRadiomics	0.720 (0.588–0.852)	63.64% (51.58%–74.19%)	65.22% (44.89%–81.19%)	62.79% (47.86%–75.62%)	0.556 (0.372–0.704)	*reference*	0.0963
	SAM-Med3D	0.562 (0.398–0.726)	63.64% (51.58%–74.19%)	43.48% (25.63%–63.19%)	74.42% (59.76%–85.07%)	0.455 (0.263–0.625)	0.0963	*reference*
	SAM-PR	**0.760** **(0.637**–**0.884)**	71.21% (59.36%–80.73%)	73.91% (53.53%–87.45%)	69.77% (54.89%–81.40%)	0.642 (0.485–0.774)	0.1105	0.0255*
SVM	PyRadiomics	0.719 (0.586–0.852)	71.21% (60.96%–82.00%)	52.17% (36.81%–74.37%)	81.40% (67.38%–90.26%)	0.591 (0.389–0.750)	*reference*	0.1445
	SAM-Med3D	0.611 (0.446–0.775)	71.21% (59.36%–80.73%)	56.52% (36.81%–74.37%)	79.07% (64.79%–88.58%)	0.578 (0.390–0.735)	0.1445	*reference*
	SAM-PR	**0.753** **(0.629**–**0.878)**	74.24% (62.57%–83.25%)	60.87% (40.79%–77.84%)	81.40% (67.38%–90.26%)	0.622 (0.444–0.773)	0.1867	0.0488*
RF	PyRadiomics	0.662 (0.516–0.808)	66.67% (54.66%–76.84%)	52.17% (32.96%–70.76%)	74.42% (59.76%–85.07%)	0.522 (0.324–0.694)	*reference*	0.846
	SAM-Med3D	0.643 (0.474–0.811)	77.27% (65.83%–85.71%)	60.87% (40.79%–77.84%)	86.05% (72.74%–93.44%)	0.651 (0.470–0.792)	0.846	*reference*
	SAM-PR	**0.708** **(0.572**–**0.844)**	75.76% (62.57%–83.25%)	60.87% (40.79%–77.84%)	83.72% (67.38%–90.26%)	0.622 (0.421–0.764)	0.255	0.4582
XGB	PyRadiomics	0.635 (0.487–0.783)	62.12% (50.06%–72.85%)	52.17% (32.96%–70.76%)	67.44% (52.52%–79.51%)	0.490 (0.293–0.655)	*reference*	0.5176
	SAM-Med3D	0.707 (0.548–0.866)	74.24% (62.57%–83.25%)	43.48% (25.63%–63.19%)	90.70% (78.40%–96.32%)	0.541 (0.333–0.706)	0.5176	*reference*
	SAM-PR	**0.766** **(0.642**–**0.891)**	69.70% (57.78%–79.45%)	69.57% (49.13%–84.40%)	69.77% (54.89%–81.40%)	0.615 (0.455–0.765)	0.0226*	0.4691
LGBM	PyRadiomics	0.645 (0.492–0.798)	62.12% (50.06%–72.85%)	56.52% (36.81%–74.37%)	65.12% (50.17%–77.58%)	0.510 (0.318–0.667)	*reference*	0.7024
	SAM-Med3D	**0.686** **(0.522**–**0.850)**	72.73% (60.96%–82.00%)	69.57% (49.13%–84.40%)	74.42% (59.76%–85.07%)	0.640 (0.462–0.783)	0.7024	*reference*
	SAM-PR	**0.686** **(0.550**–**0.821)**	62.12% (50.06%–72.85%)	43.48% (25.63%–63.19%)	72.09% (57.31%–83.25%)	0.444 (0.250–0.622)	0.4879	0.9958
Human	Doctor #1	**/**	66.67% (54.66%–76.84%)	26.09% (12.55%–46.47%)	88.37% (75.52%–94.93%)	0.353 (0.138–0.550)	/	/
	Doctor #2	**/**	68.18% (56.21%–78.15%)	30.43% (15.60%–50.87%)	88.37% (75.52%–94.93%)	0.400 (0.182–0.595)	/	/

LR, logistic regression; SVM, support vector machine; RF, random forest; XGB, extreme gradient boosting; LGBM, light gradient-boosting machine, SAM-PR, the fusion feature set with features extracted by both PyRadiomics and SAM-Med3D extractors.

*p*-values were calculated by Delong test. *p*-values are unadjusted for multiplicity.

The bold value means it is statistically significant.

*p-value indicates statistically significant.

#### 3.3.3 AI classifier vs. human doctor

While real-world staging requires a comprehensive clinical and endoscopic context, restricting human evaluators to the identical input features utilized by the model (MRI, age, sex) was a deliberate design choice. This isolated the diagnostic yield of the imaging modality, ensuring a controlled comparison of visual interpretation vs. high-dimensional feature extraction without introducing confounding clinical variables. Both experts demonstrated a strong ability to correctly identify early-stage disease [specificity of 88.37% (95% CI: 75.52%–94.93%) and 88.37% (95% CI: 75.52%–94.93%), respectively], however a relatively limited accuracy in identifying Stage IVa cases [sensitivity of 26.09% (95% CI: 12.55%–46.47%) and 30.43% (95% CI: 15.60%–50.87%), respectively]. In comparison, our optimal model (XGB-SAM-PR) demonstrated a more balanced diagnostic performance, with the specificity of 69.77% (95% CI: 54.89%–81.40%) and a sensitivity of 69.57 (95% CI: 49.13%–84.40%). Consequently, the AI model achieved a substantially higher F1-score compared to the two human evaluators (0.615 (95% CI: 0.455–0.765) vs. 0.353 (95% CI: 0.138–0.550) and 0.615 (95% CI: 0.455–0.765) vs. 0.400 (95% CI: 0.182–0.595), respectively). However, McNemar's test showed no significant differences in classification performance between all the five fusion models and the two independent human readers (*p*-values ranging from 0.267 to 1.000, Supplementary Table S2).

### 3.4 Model interpretability using SHAP analysis

The LR and SVM models identified T1WI_wavelet-HHH_glcm_DifferenceEntropy as the most impactful feature (Supplementary Figures S1A,B). In contrast, the tree-based models (RF and XGB) both converged on T2WI_log-sigma-3-0-mm-3D_glcm_Imc1 as the top predictor (Supplementary Figures S1C,D). Notably, the RF model almost exclusively prioritized traditional radiomics features, placing seven out of the eight SAM-Med3D features at the bottom of the importance list. This lack of hybrid integration likely explains why the RF-based fusion model underperformed relative to the other algorithms in this task. Conversely, the SVM and XGB models demonstrated better hybrid data integration. The SVM model ranked T1WI_sam_feat_138 and CE-T1WI_sam_feat_39 within its top seven most important features, alongside top-ranked radiomics features. Similarly, the XGB model identified CE-T1WI_sam_feat_152 as its fifth most predictive feature, placing it above the other five radiomics predictors.

## 4 Discussion

This study introduces a domain-specific hybrid framework for NPC staging and presents several significant findings. First, while previous Transformer-based studies in NPC have predominantly relied on feature extractors pre-trained on 2D, non-medical datasets, we demonstrated the translational value of SAM-Med3D—a voxel-centric ViT explicitly pre-trained on over 140,000 volumetric medical masks. By capturing global anatomical dependencies rather than relying on the local receptive fields of traditional CNNs, the SAM-Med3D features alone extracted reliable and robust representations for stage classification. It provides a substantial capability to extract reliable and robust DL features from MRI scans of NPC patients for stage classification using XGB classifier (AUC = 0.707, Table 2), even without disease-specific fine-tuning. Second, our analysis revealed that hand-crafted PyRadiomics features, and ViT-derived features offer complementary diagnostic information. While integrating SAM-Med3D and PyRadiomics features yielded a statistically moderate AUC improvement over unimodal approaches, its practical utility is significant when evaluating diagnostic balance. The primary clinical risk in NPC staging is the under-treatment of advanced disease. Unimodal pipelines struggled to capture these cases; for instance, the SAM-Med3D LR model exhibited a high specificity (74.42%) but critically a low sensitivity (43.48%) for Stage IVa disease. By integrating global 3D spatial context with sub-visual textural heterogeneity, the fusion model (LR-SAM-PR) explicitly resolved this diagnostic blind spot, improving sensitivity to 73.91% and achieving an optimal F1-score of 0.642. Clinically, this hybrid framework acts as a more balanced tool, successfully capturing high-risk advanced disease that unimodal models and human readers frequently overlook. Finally, our optimal model (LR-SAM-PR) demonstrated a diagnostic performance that was not only comparable but, regarding F1-score, potentially better than that of individual otolaryngologists assessing MRI scans in isolation. The experts achieved high specificity (88.37%) but low sensitivity (26.09%–30.43%) for Stage IVa disease. Stage IVa is defined by extensive local invasion or advanced nodal involvement. While early-stage lesions are visually distinct, subtle micro-invasions into the skull base or borderline nodal metastases could be ambiguous on standard MRI sequences. In contrast, the LR-SAM-PR model achieved a much more balanced diagnostic profile (Sensitivity: 73.91%, Specificity: 69.77%). This balance is driven by the synergistic integration of multi-scale features. While PyRadiomics objectively quantifies sub-visual, voxel-level textural heterogeneity, the SAM-Med3D embeddings maintain the global 3D spatial context of the tumor within the complex nasopharyngeal anatomy. By relying on these high-dimensional mathematical representations, the fusion model could provide a highly sensitive and balanced assessment of advanced-stage disease. This suggests that in clinical scenarios where a single physician interprets imaging without multidisciplinary consultation, the AI model could serve as a reliable “second opinion”, potentially reducing the rate of under-staging.

The AI radiomic applications among NPC patients staging were widely explored these years, from typical machine learning approaches to novel DL approaches, targeting multiple tasks including diagnosis, staging, treatment decisions, therapeutic efficacy, long-term prognosis prediction etc. (17). However, most of these NPC DL related studies have revolved around CNN. While effective, CNNs are inherently limited by their local receptive fields, potentially missing long-range spatial dependencies within the MRI volume.

In recent years, ViT have emerged as a powerful alternative to CNNs, leveraging self-attention mechanisms to process image patches as sequences and capture global context (9). Nevertheless, the application of ViTs in NPC remains under-investigated, primarily due to the scarcity of large-scale datasets and the high cost of acquiring high-quality clinical data (17). While recent studies have explored Transformer-based architectures, they face notable limitations. For instance, Han et al. (18), Hou et al. (19), and Song et al. (20) employed Swin-Transformers for tasks ranging from segmentation to prognostic radiopathomics and T-stage classification. A critical limitation of these studies is the feature extractors. Swin-Transformers models were pre-trained on ImageNet—a non-medical dataset—which may significantly constrain performance. Additionally, while Li et al. incorporated attention mechanisms for adaptive therapy prediction, this module was restricted to feature fusion rather than serving as the primary feature extractor (21).

In contrast, our study has several methodological strengths. Firstly, this is the first study to evaluate a ViT-based medical image-specific foundation model (SAM-Med3D) for MRI interpretation in NPC. SAM-Med3D is a voxel-centric vision foundation model developed specifically for volumetric medical image analysis. It is pre-trained on the largest volumetric medical dataset, comprising over 140,000 masks across diverse modalities including MRI and CT. Additionally, unlike previous 2D, SAM-Med3D employs a holistic 3D Transformer architecture incorporating 3D positional encodings and an attention block, which ensures superior transferability to downstream clinical tasks such as NPC interpretation. Secondly, we innovatively implemented a fusion strategy by integrating hand-crafted radiomic features with ViT-derived deep features. This approach is supported by a recent systematic review, which demonstrated that combining hand-crafted and deep learning features improved diagnostic performance in 74% of studies (22). However, 68/69 of the studies relied exclusively on the CNN algorithm. Unlike ViTs, CNNs possess inherent limitations in capturing global image characteristics due to their restricted local receptive fields. Lastly, acknowledging that different machine learning algorithms possess distinct inductive biases and may respond differently to various feature representations, we employed five different classifiers for the final model construction. This comprehensive comparative analysis allowed us to systematically evaluate the compatibility of our feature sets with diverse algorithmic architectures, ensuring the identification of the optimal feature-classifier combination for accurate NPC staging.

Despite these promising results, our study has limitations. First, the analysis was conducted on a single-center cohort without external validation, which limits the generalizability of the findings. First, the analysis was conducted on a single-center cohort without external validation, which limits the generalizability of the findings. However, our model evaluation was performed on a strictly independent, hold-out validation set (*n* = 66), comprising 25% of the total cohort, which the models never encountered during training. We also applied LASSO with 5-fold stratified cross-validation explicitly within the training set. This reduced the hundreds of extracted features down to a highly constrained, compact signature (17 features for the SAM-PR fusion set) prior to any model construction. To further fortify the study's internal validity and mathematically address potential performance inflation due to random data partitioning, we executed a rigorous 1,000-iteration optimism-corrected bootstrap analysis on our optimal LR classifier. This internal validation yielded an apparent AUC of 0.849 with an exceptionally negligible mean optimism of 0.013, resulting in a robust, overfitting-adjusted AUC of 0.8356. Complementing this, our learning curve analysis demonstrated classic statistical convergence between the training and cross-validation scores at the maximum sample size, with clear plateauing that signifies early data saturation. This empirical evidence confirms that our cohort size is statistically sufficient for the model's feature capacity and entirely free from high-variance over-parameterization. Besides, we believe our current cohort inherently contains a degree of multi-center-like technical variance. As detailed in our methodology, the MRI data were acquired using different magnetic field strengths and distinct hardware architectures (GE Discovery MR750w 3.0T and Philips Achieva 1.5T systems). To manage this, we applied rigorous standardization, calibration, and intensity normalization protocols. The fact that our fusion model maintained strong discriminative ability across this hardware variance demonstrates a degree of technical robustness. Second, we utilized the pre-trained SAM-Med3D strictly as a zero-shot feature extractor. To investigate the potential of task-specific adaptation, we conducted a secondary experiment utilizing Low-Rank Adaptation (LoRA) to fine-tune the SAM-Med3D encoder on our training cohort (*n* = 198). However, evaluating the LoRA-adapted fusion features (SAM-PR) on the independent validation set revealed a consistent degradation in performance across all algorithms (e.g., LR AUC decreased from 0.760 to 0.697, Supplementary Figure S2). This indicates that despite drastically reducing trainable parameters, applying LoRA on a constrained sample size causes the model to overfit to the local training distribution. Consequently, our findings empirically demonstrate that relying on the frozen, zero-shot embeddings provides a more robust and generalizable feature space for NPC staging in cohorts of this size. Third, while our fusion approach (SAM-PR) consistently yielded numerically higher AUCs and F1-scores than individual feature sets across all five machine learning algorithms, the statistical comparisons must be interpreted with caution. Because multiple pairwise comparisons were conducted across the models (Table 2), there is an inherent risk of Type I errors. When a False Discovery Rate (FDR) correction (Benjamini-Hochberg procedure) is applied to these comparisons, the adjusted *p*-values exceed the 0.05 threshold of significance. In the context of our relatively small independent validation cohort (*n* = 66), strict multiplicity corrections severely limit statistical power. Therefore, our findings should be viewed as exploratory rather than confirmatory. The primary value of our results lies in the consistent directional improvement observed when integrating SAM-Med3D features with traditional radiomics. Additionally, although the SAM-PR models consistently yielded numerically higher F1 scores than human readers, McNemar's test revealed no statistically significant difference in performance. This suggests that while the models are competitive, current sample sizes may be insufficient to establish definitive superiority. Future studies utilizing larger, multi-center datasets would not only provide the statistical power necessary to validate these trends but also enable end-to-end fine-tuning, potentially further elevating diagnostic accuracy. Finally, due to the skewness of the staging data, we binarized the outcome into Stage ≤ III vs. Stage IVa. While differentiating between these two cohorts represents a critical prognostic and therapeutic tipping point in NPC management. We acknowledge that distinguishing between early stages is also clinically vital. Future work should aim to evaluate more granular, multi-class staging tasks, such as predicting specific T- and N-stages or long-term survival outcomes, once larger, multi-center cohorts with sufficient event rates are available.

## 5 Conclusions

The integrated model, combining conventional radiomics with SAM-Med3D features, consistently showed higher accuracy than its unimodal counterparts across five distinct algorithms for NPC stage I, II, III and stage IVa differentiation. All five fusion models demonstrated comparable discriminative ability compared to experienced experts. These findings suggest that hybrid feature frameworks can serve as powerful objective tools in NPC staging.

## Data Availability

The original contributions presented in the study are included in the article/Supplementary Material, further inquiries can be directed to the corresponding author/s.
