# Supplementary material for: Augmenting medical image specific foundation model with classical radiomic signatures for improved nasopharyngeal carcinoma stage classification
Source: Front Med Technol. 2026 Jul 15;8:1863203. doi: 10.3389/fmedt.2026.1863203 (PMC13415764; doi:10.3389/fmedt.2026.1863203)
Supplement: Supplementary file 1 [file Table1.docx]

**Supplementary Figure 1. SHAP analysis**

These four figure illustrate the most influential features contributing to the classification of NPC stage (Stage ≤III vs. Stage IVa) across four different machine learning algorithms: (A) Logistic Regression, (B) Support Vector Machine (SVM), (C) Random Forest (RF), and (D) XGBoost (XGB). Feature importance is quantified by the mean absolute SHAP value (x-axis), representing the average magnitude of impact a feature has on the model's output prediction. Features are ranked in descending order of importance; longer bars indicate a greater contribution to the model's decision-making process.

**Supplementary Figure 1A**


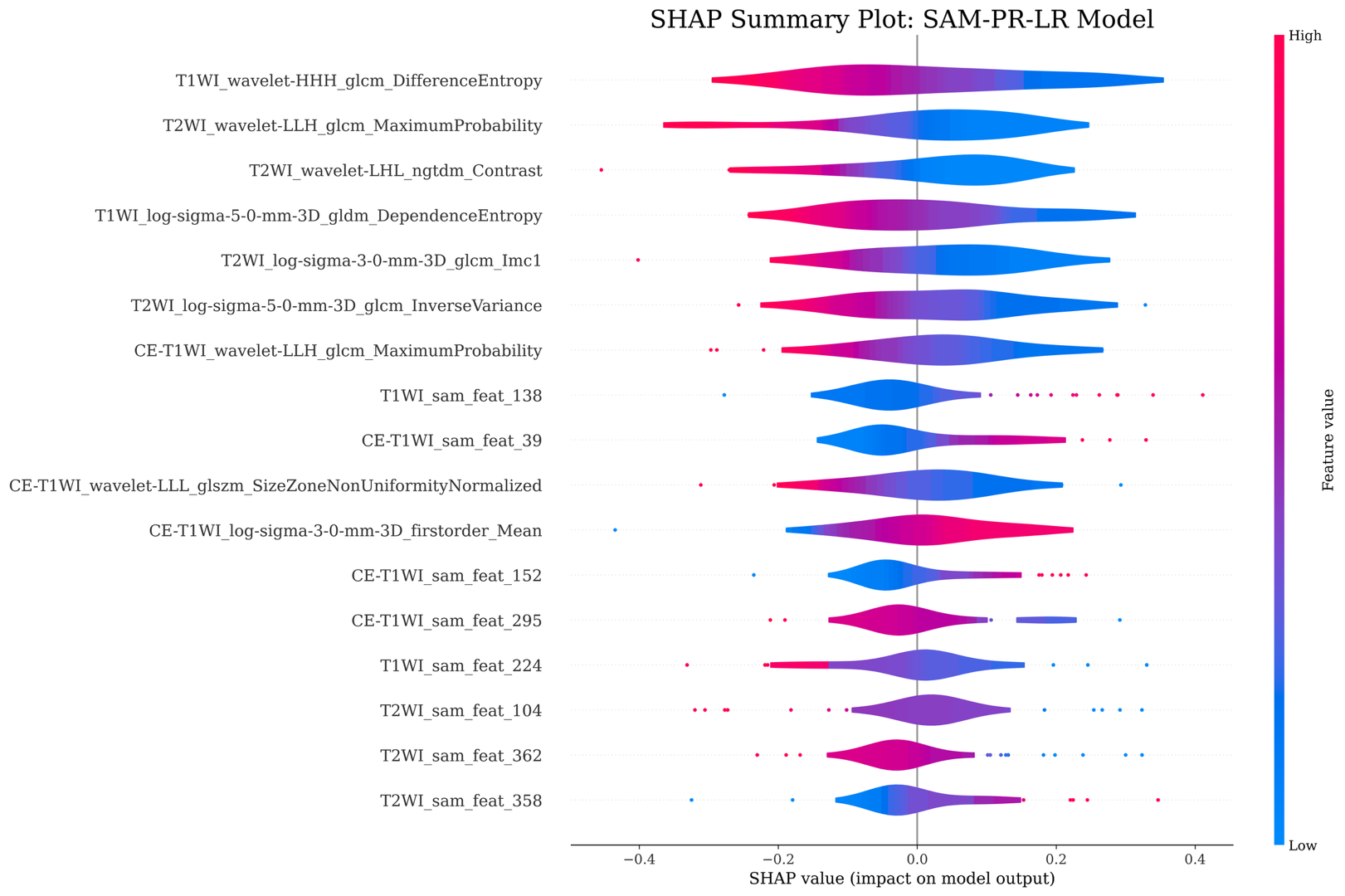


**Supplementary Figure 1B**


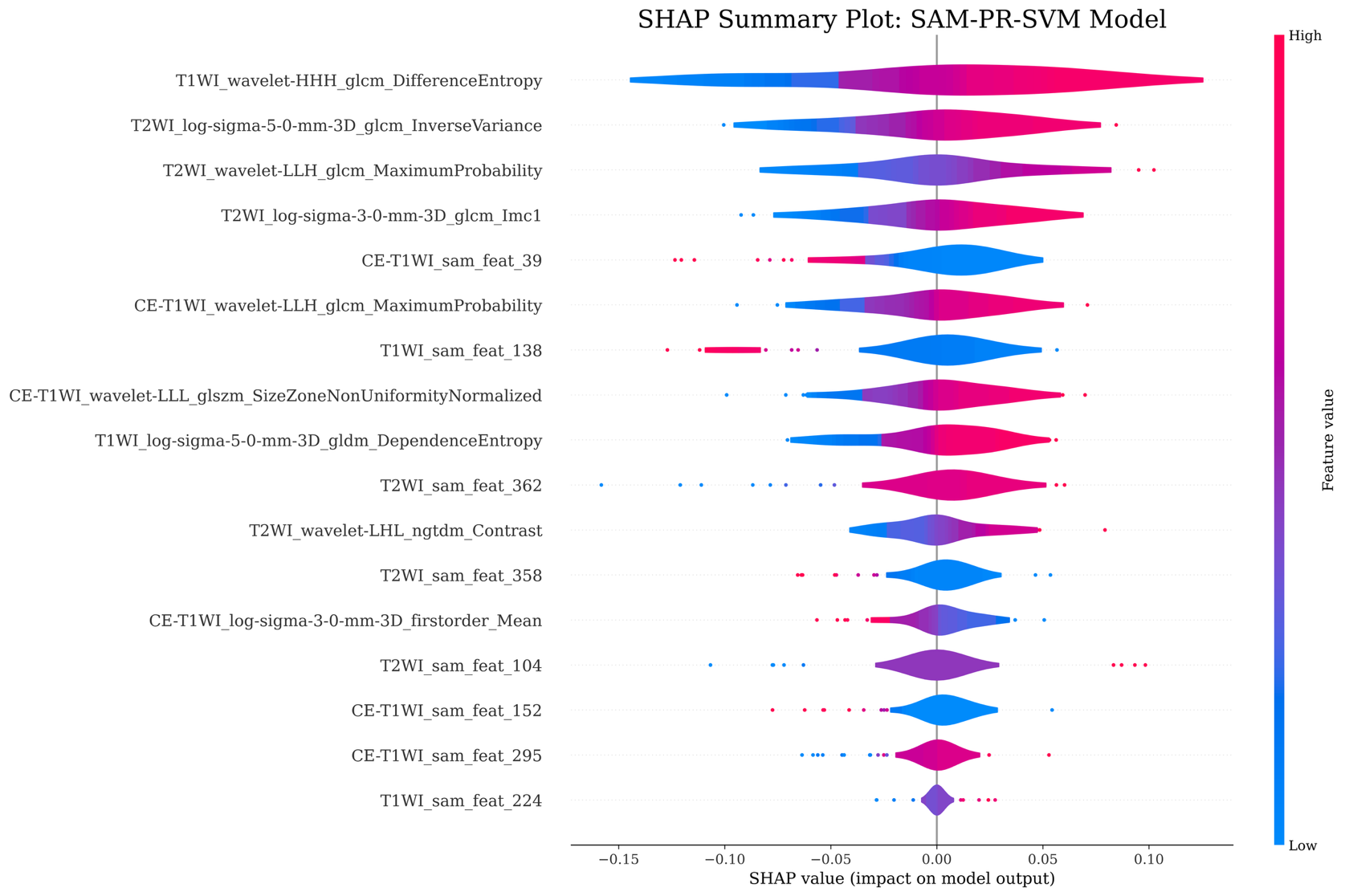


**Supplementary Figure 1C**


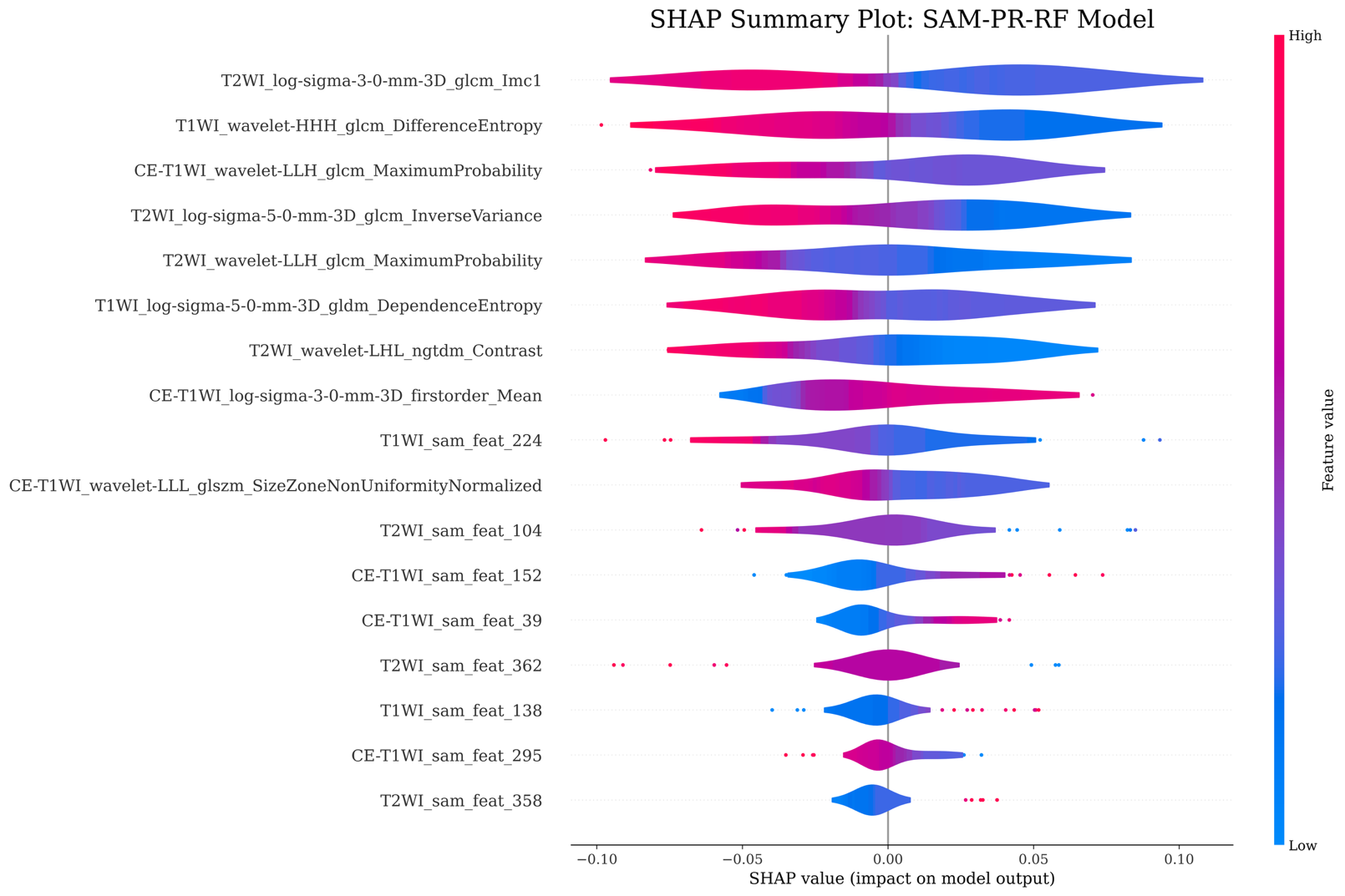


**Supplementary Figure 1D**


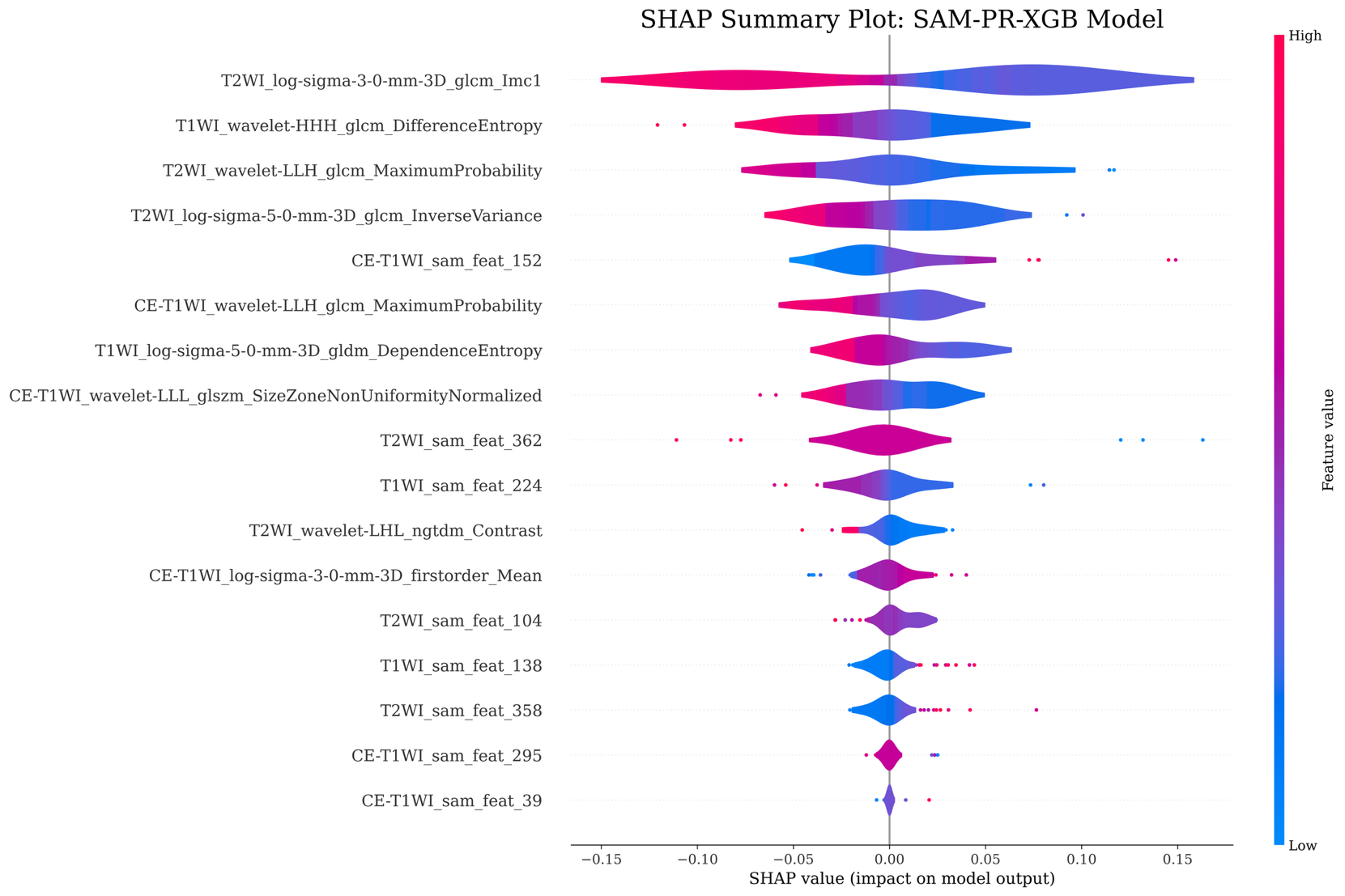
**Supplementary Figure 2. All algorithms on SAM-PR (LoRA-fine-tuned)**


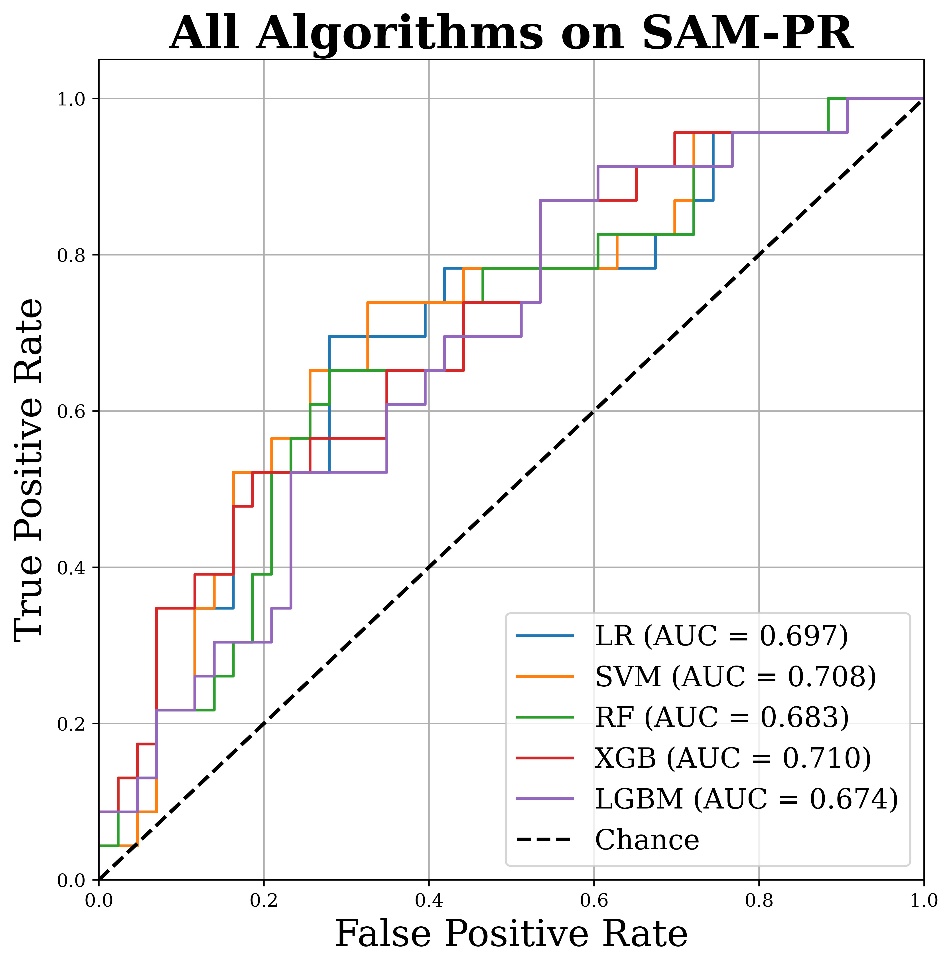


Comparative performance of the five algorithms utilizing the optimal Fusion (SAM-PR) feature set.

LoRA, Low-Rank Adaptation

**Supplementary Figure 3. Learning curve**


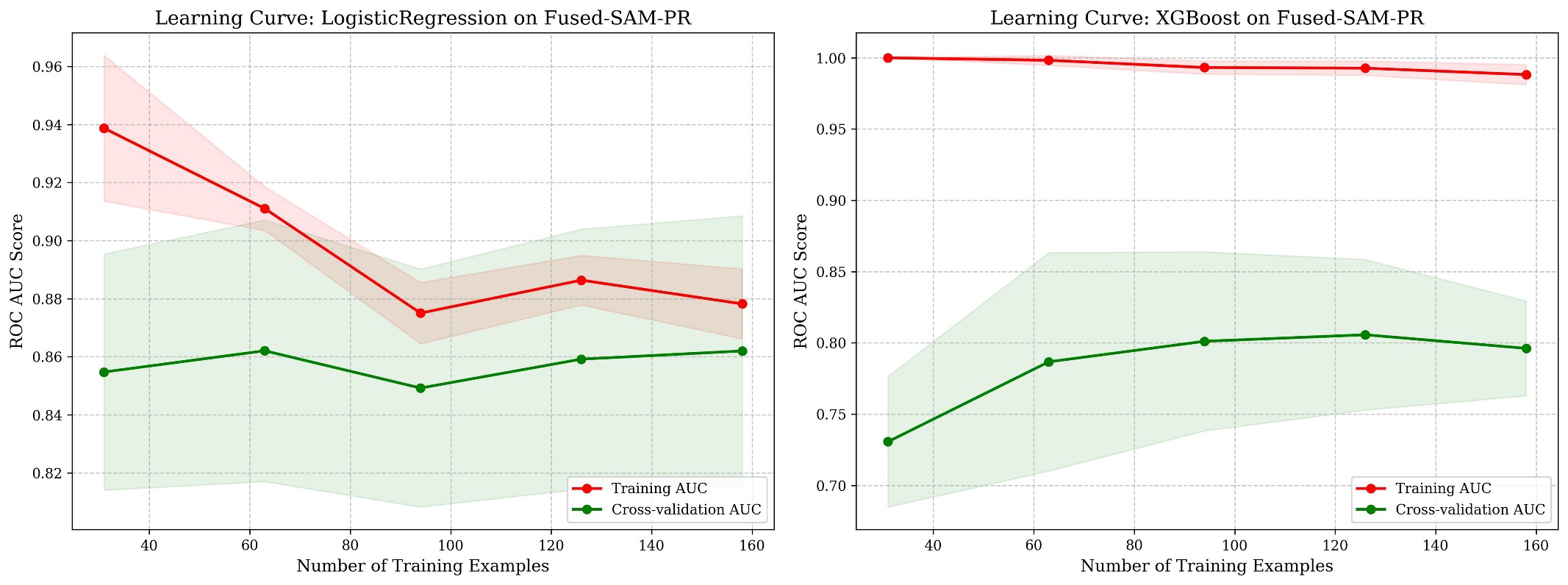


| **Supplementary Table 1. Feature sets description** | | | |  |
| --- | --- | --- | --- | --- |
| **Feature set** | **T1WI** | **T2WI** | **CE-T1WI** | **Clinical features** |
| PyRadiomics | T1WI_original_firstorder_Energy; T1WI_wavelet-HHH_glcm_DifferenceEntropy; T1WI_log-sigma-5-0-mm-3D_gldm_DependenceEntropy | T2WI_wavelet-LLH_glcm_MaximumProbability; T2WI_wavelet-LHL_ngtdm_Contrast; T2WI_log-sigma-3-0-mm-3D_glcm_Imc1; T2WI_log-sigma-5-0-mm-3D_glcm_InverseVariance; T2WI_log-sigma-5-0-mm-3D_gldm_LargeDependenceEmphasis | CE-T1WI_wavelet-LLH_glcm_MaximumProbability; CE-T1WI_wavelet-LHH_gldm_DependenceNonUniformityNormalized; CE-T1WI_wavelet-LLL_glszm_SizeZoneNonUniformityNormalized; CE-T1WI_log-sigma-3-0-mm-3D_firstorder_Mean | / |
| SAM-Med3D | T1WI_sam_feat_14; T1WI_sam_feat_19; T1WI_sam_feat_43; T1WI_sam_feat_120; T1WI_sam_feat_133; T1WI_sam_feat_157; T1WI_sam_feat_165; T1WI_sam_feat_178; T1WI_sam_feat_224; T1WI_sam_feat_254; T1WI_sam_feat_279; T1WI_sam_feat_331 | T2WI_sam_feat_75; T2WI_sam_feat_78; T2WI_sam_feat_104; T2WI_sam_feat_176; T2WI_sam_feat_254; T2WI_sam_feat_256; T2WI_sam_feat_289; T2WI_sam_feat_343; T2WI_sam_feat_358; T2WI_sam_feat_362 | CE-T1WI_sam_feat_3; CE-T1WI_sam_feat_39; CE-T1WI_sam_feat_63; CE-T1WI_sam_feat_81; CE-T1WI_sam_feat_86; CE-T1WI_sam_feat_96; CE-T1WI_sam_feat_105; CE-T1WI_sam_feat_185; CE-T1WI_sam_feat_194; CE-T1WI_sam_feat_205; CE-T1WI_sam_feat_219; CE-T1WI_sam_feat_251; CE-T1WI_sam_feat_285; CE-T1WI_sam_feat_297; CE-T1WI_sam_feat_333; CE-T1WI_sam_feat_338; CE-T1WI_sam_feat_352 | age; gender_male |
| SAM-PR | T1WI_wavelet-HHH_glcm_DifferenceEntropy; T1WI_log-sigma-5-0-mm-3D_gldm_DependenceEntropy; T1WI_sam_feat_138; T1WI_sam_feat_224 | T2WI_wavelet-LLH_glcm_MaximumProbability;T2WI_wavelet-LHL_ngtdm_Contrast; T2WI_log-sigma-3-0-mm-3D_glcm_Imc1; T2WI_log-sigma-5-0-mm-3D_glcm_InverseVariance; T2WI_sam_feat_104; T2WI_sam_feat_358; T2WI_sam_feat_362 | CE-T1WI_wavelet-LLH_glcm_MaximumProbability; CE-T1WI_wavelet-LLL_glszm_SizeZoneNonUniformityNormalized; CE-T1WI_log-sigma-3-0-mm-3D_firstorder_Mean; CE-T1WI_sam_feat_39; CE-T1WI_sam_feat_152; CE-T1WI_sam_feat_295 | / |
| glcm, gray level co-occurrence matrix; gldm, gray level dependence matrix; glszm, gray level size zone matrix; ngtdm, neighbouring gray tone difference matrix | | | |  |

| **Supplementary Table 2. McNemar’s test between models and human readers** | | | |
| --- | --- | --- | --- |
|  |  | **Human readers** | |
|  |  | vs. Doctor #1 (p-value) | vs. Doctor #2 (p-value) |
| **Models** | LR-SAM-PR | 0.664 | 0.824 |
|  | SVM-SAM-PR | 0.267 | 0.388 |
|  | RF-SAM-PR | 0.332 | 0.454 |
|  | XGB-SAM-PR | 0.824 | 1.000 |
|  | LGBM-SAM-PR | 0.629 | 0.454 |
| LR, logistic regression; SVM, support vector machine; RF, random forest; XGB, extreme gradient boosting; LGBM, light gradient-boosting machine, SAM-PR, the fusion feature set with features extracted by both PyRadiomics and SAM-Med3D extractors.  p-values were calculated by McNemar’s test. | | | |

| **Supplementary Table 3. Optimism-Corrected AUC of different models based on three feature sets** | | | | |
| --- | --- | --- | --- | --- |
| **Algorithm** | **Feature set** | **Optimism-corrected AUC** | **Apparent AUC** | **Mean optimism** |
| LR | PyRadiomics | 0.800 | 0.808 | 0.009 |
|  | SAM-Med3D | 0.789 | 0.860 | 0.071 |
|  | SAM-PR | 0.836 | 0.849 | 0.013 |
| SVM | PyRadiomics | 0.799 | 0.809 | 0.011 |
|  | SAM-Med3D | 0.781 | 0.856 | 0.075 |
|  | SAM-PR | 0.835 | 0.852 | 0.016 |
| RF | PyRadiomics | 0.947 | 1.000 | 0.053 |
|  | SAM-Med3D | 0.806 | 0.887 | 0.081 |
|  | SAM-PR | 0.967 | 1.000 | 0.033 |
| XGB | PyRadiomics | 0.937 | 1.000 | 0.064 |
|  | SAM-Med3D | 0.794 | 0.887 | 0.093 |
|  | SAM-PR | 0.887 | 0.961 | 0.075 |
| LGBM | PyRadiomics | 0.934 | 1.000 | 0.066 |
|  | SAM-Med3D | 0.821 | 0.918 | 0.097 |
|  | SAM-PR | 0.947 | 1.000 | 0.053 |
| LR, logistic regression; SVM, support vector machine; RF, random forest; XGB, extreme gradient boosting; LGBM, light gradient-boosting machine, SAM-PR, the fusion feature set with features extracted by both PyRadiomics and SAM-Med3D extractors.  p-values were calculated by Delong test. p-values are unadjusted for multiplicity. | | | | |
